# PACAP Is Lethal to *Flavobacterium psychrophilum* Through Either Direct Membrane Permeabilization or Indirectly, by Priming the Immune Response in Rainbow Trout Macrophages

**DOI:** 10.3389/fimmu.2019.00926

**Published:** 2019-04-26

**Authors:** Shawna L. Semple, Tania Rodríguez-Ramos, Yamila Carpio, John S. Lumsden, Mario P. Estrada, Brian Dixon

**Affiliations:** ^1^Department of Biology, University of Waterloo, Waterloo, ON, Canada; ^2^Center for Genetic Engineering and Biotechnology, Havana, Cuba; ^3^Department of Pathobiology, Ontario Veterinary College, University of Guelph, Guelph, ON, Canada

**Keywords:** rainbow trout, antimicrobial peptide, PACAP, RTS11, cytokines, fish pathogen, *Flavobacterium psychrophilum*

## Abstract

Pituitary adenylate cyclase-activating polypeptide (PACAP) is a multifunctional neuropeptide that is widely distributed in mammals and is capable of performing roles as a neurotransmitter, neuromodulator, and vasodilator. This polypeptide belongs to the glucagon/secretin superfamily, of which some members have been shown to act as antimicrobial peptides in both mammalian and aquatic organisms. In teleosts, PACAP has been demonstrated to have direct antimicrobial activity against several aquatic pathogens, yet this phenomenon has never been studied throughout a live bacterial challenge. The present study focuses on the influence of synthetic *Clarias gariepinus* 38 amino acid PACAP on the rainbow trout monocyte/macrophage-like cell line, RTS11, when exposed to the coldwater bacterial pathogen *Flavobacterium psychrophilum*. PACAP was shown to have direct antimicrobial activity on *F. psychrophilum* when grown in both cytophaga broth and cell culture media (L-15). Further, the ability of teleostean PACAP to permeabilize the membrane of an aquatic pathogen, *F. psychrophilum*, was demonstrated for the first time. The viability of RTS11 when exposed to PACAP was also observed using a trypan blue exclusion assay to determine optimal experimental doses of the antimicrobial peptide. This displayed that only concentrations higher than 0.1 μM negatively impacted RTS11 survival. Interestingly, when RTS11 was pre-treated with PACAP for 24 h before experiencing infection with live *F. psychrophilum*, growth of the pathogen was severely inhibited in a dose-dependent manner when compared to cells receiving no pre-treatment with the polypeptide. Relative expression of pro-inflammatory cytokines (IL-1β, TNFα, and IL-6) and PACAP receptors (VPAC1 and PAC1) was also analyzed in RTS11 following PACAP exposure alone and in conjunction with live *F. psychrophilum* challenge. These qRT-PCR findings revealed that PACAP may have a synergistic effect on RTS11 immune function. The results of this study provide evidence that PACAP has immunostimulatory activity on rainbow trout immune cells as well as antimicrobial activity against aquatic bacterial pathogens such as *F. psychrophilum*. As there are numerous pathogens that plague the aquaculture industry, PACAP may stimulate the teleost immune system while also providing an efficacious alternative to antibiotic use.

## Introduction

Due to the rising demand for fish protein (1), aquaculture has become a necessary means to protect wild populations from irreversible overfishing. As such, it is imperative that these culture systems have a minimal impact on the environment while still being able to provide the high-quality product for market. To attain this goal, alternative methods must be developed to combat infectious disease as this is one of the greatest sources of instability and financial cost in aquaculture. Global losses due to aquatic infections total approximately $6 billion USD (2) and currently, fish farmers have few methods outside of antibiotics to prevent/control outbreaks. With multi-drug resistance continually rising [reviewed by Watts et al. (3), Santos and Ramos (4)], antibiotic use in aquaculture is tightly regulated which often leaves farmers with few options when outbreaks do occur. This problem has led to an increased interest in the development of alternative approaches for disease prevention, including the use of naturally occurring antimicrobial peptides (AMPs).

Antimicrobial peptides are a diverse class of highly conserved molecules that are produced as a first line of defense in multicellular organisms. These small peptides (12–50 amino acids) are essential components of innate immunity capable of antimicrobial activity against a wide range of microbial pathogens [reviewed by Zhang and Gallo (5)], which notably includes multi-drug resistant isolates (6, 7). Most AMPs are cationic amphipathic peptides that function by attacking the negatively charged membranes of microorganisms [reviewed by Mahlapuu et al. (8)]. Based on their secondary structures, AMPs can be characterized as one of four types, β-sheet, α-helix, extended and loop with β-sheet and α-helix being the most common [reviewed by Bahar and Ren (9)]. Functionally, they can be characterized as either membrane disruptive AMPs, causing membrane permeabilization, or nonmembrane disruptive AMPs, which directly passage into cells and act on intracellular targets [reviewed by Kang et al. (10)]. Besides direct destruction of pathogens, AMPS also perform immunomodulatory functions in higher vertebrates [reviewed by Otvos (11)] and as a result are also called “host defense peptides” (HDPs) to emphasize these additional activities. The potential immunomodulatory effects are diverse including stimulation of chemotaxis, immune cell differentiation, initiation of adaptive immunity and stimulation of both pro- and anti- inflammatory cytokines (12–15). Though novel AMPs and their activities are continuously being discovered, one that has gained a lot of interest as a result of its vast pleiotropic effects is pituitary adenylate cyclase activating polypeptide (PACAP).

Initially, PACAP was discovered as a neuropeptide due to its ability to stimulate adenylate cyclase activity in ovine pituitary cell cultures (16). Derived from a 175 amino acid precursor, functional PACAP has two molecular forms. The first has 38 amino acids (PACAP-38) while the other form is truncated containing only 27 residues [PACAP-27, (17, 18)]. Of the two, PACAP-38 is considered to be more bioactive as it has been shown to display 100–1000 times greater potency in stimulating cell proliferation, DNA synthesis and inositol phospholipid turnover in cells (19, 20). Further analysis of PACAP-38 revealed that this peptide shared 68% sequence similarity with vasoactive intestinal polypeptide (VIP), thereby classifying PACAP as a member of the secretin/glucagon/growth hormone-releasing hormone/vasoactive intestinal peptide superfamily (16). As this was the case, it is not surprising that PACAP-38 is able to bind with equal affinity to the same G-coupled protein receptors (GCPRs) as VIP, vasoactive intestinal polypeptide receptor 1 (VPAC1) and vasoactive intestinal polypeptide receptor 2 (VPAC2), while also binding to its own receptor, pituitary adenylate cyclase-activating polypeptide type I receptor [PAC1, (21, 22)]. All three of these receptors have a wide tissue distribution much like the neuropeptides themselves (21, 23, 24). PACAP-38 in particular, displays a broad range of functions in multiple tissue types, including antimicrobial activity, growth, immunomodulation, neural development, anti-tumor activity and metabolism to name a few (25–29). From an evolutionary perspective, the amino acid sequence of PACAP-38 is identical in all mammals with only a few amino acid substitutions when comparing to other species (e.g., frog, salmon, tunicate, etc.). PACAP-38 therefore must play a vital role in physiological function as it has remained essentially unchanged for ~700 million years (30). This broad functional profile as well as its highly conserved nature has made PACAP-38 an attractive candidate for disease control and therapeutic use in aquaculture.

Though there are numerous pathogens that impact the aquaculture industry, *F. psychrophilum* has proven to be a global threat in the culture of freshwater rainbow trout (*Oncorhynchus mykiss*). This gram-negative bacterial pathogen is the causative agent of bacterial coldwater disease (BCWD) and rainbow trout fry syndrome (RTFS), two separate conditions that can occur depending on the bacterial isolate, geographical location and age of the host (31). These conditions present as either an acute bacteremia primarily in small fish (RTSF) or as a more chronic disease most commonly characterized by an ulcerative dermatitis (BCWD) in larger fish (31, 32). Though variable, mortality resulting from these conditions without intervention generally ranges from 2–30% (33) but in extreme cases can be as high as 50–90% (34–36). Despite concerted efforts to selectively breed for resistance to *F. psychrophilum* (33, 37–39) and multiple attempts to develop an effective vaccine [reviewed by Gomez et al. (40), Makesh et al. (41), and Hoare et al. (42)], there is little information regarding the pathogenesis of the organism. Based on the data presented thus far, it appears that *F. psychrophilum* has an intricate relationship with the spleen and head kidney macrophages of rainbow trout (43, 44). As such, the spleen monocyte/macrophage-like cell line, RTS11 (45), would be an ideal model system for studying *F. psychrophilum* infections. As a relevant immune cell line, RTS11 could provide further insight regarding the immunomodulatory effects of PACAP-38 as well as its antimicrobial function within an appropriate infection model.

Previous research involving teleostean PACAP-38 has focused on assessing its antimicrobial activity when directly dosing aquatic pathogens (46), the growth/immunomodulatory effects of the peptide alone (47–49), or how viral/bacterial infection can influence gene expression of the peptide and its associated receptors (50). Though these results were promising, there is yet to be a study evaluating the activity of PACAP-38 in a live infection model. Furthermore, the effect of PACAP-38 has never been explored with respect to the industrially relevant pathogen, *F. psychrophilum*. The purpose of this study was to measure and understand the antimicrobial activity of PACAP-38 on *F. psychrophilum* as well as to determine whether PACAP could stimulate a protective immune response in RTS11 cells. Confirming the efficacy of PACAP in an *in vitro* infection model will provide further evidence to support its use in *in vivo* experiments. Additionally, the results of this work could provide valuable insights regarding the efficacy of PACAP-38 during live infections and thus aid in the development of a potential alternative for antibiotic use in aquaculture.

## Materials and Methods

### Maintenance of RTS11

The rainbow trout monocyte/macrophage-like cell line, RTS11 (45), was maintained as described previously by Sever et al. (51).

### Peptides

#### Synthetic PACAP From the Teleost *C. gariepinus*

*Clarias gariepinus* synthetic PACAP-38 (amino acid sequence of HSDGIFTDSYSRYRKQMAVKKYLAAVLGRRYRQRFRNK, MW of 4.7 kDa) was purchased from CS Bio (Shanghai) Ltd, China with 85% purity.

#### Synthetic HSP70 Peptide Fragment From Rainbow Trout

A synthetic peptide fragment of rainbow trout HSP70 (amino acid sequence of CGDQARTSSGASSQ, MW of 1.3 kDa) was purchased from Biomatik with 98% purity.

### Growth of *F. psychrophilum*

*Flavobacterium psychrophilum* strain 101 (FPG101) was grown as described previously by Semple et al. (44) with minor adjustments. This bacterial isolate has been characterized as virulent in experimental trials by Jarau et al. (52). Briefly, subcultures of FPG101 glycerol stocks were grown on cytophaga agar (CA) at 14°C and checked for purity. An isolated colony was then used to inoculate 3 mL of cytophaga broth (CB) and grown at 14°C for 72 h. After this time, the OD_600_ of the bacterial growth was consistently between 0.4 and 0.5, indicating a viable bacterial count of 2–5 × 10^8^ CFU/mL. For every culture of FPG101, a standard plate count (SPC) was completed to confirm the anticipated bacterial concentration.

### Minimum Inhibitory Concentration (MIC) of *C. gariepinus* PACAP-38 on *F. pyschrophilum*

The MIC of *C. gariepinus* PACAP-38 on FPG101 was assessed by a broth microdilution peptide assay (BMPA) (53). To prepare FPG101 for this assay, 3 mL of CB was inoculated with a single colony and allowed to grow overnight at 14°C. After this time, 1 mL of the growth was centrifuged at 5,000 rpm for 5 min, the supernatant removed, and the pellet resuspended in 4 mL of fresh CB, to have an OD_600_ of 0.1-0.4. Finally, the bacterial suspension was diluted in CB to obtain a final OD of 0.001.

The BMPA was made using a flat-bottom 96-well plate (Fisher Scientific). The plate set up consisted of wells containing 90 μL of bacterial suspension and 10 μL of PACAP at 10 different final concentrations from 5 to 50 μM. In the positive control wells, PACAP was substituted with 10 μL of CB while the negative control wells contained 100 μL of CB only. All PACAP concentrations and controls were tested in triplicates. The bacterial growth was monitored after 3 days of incubation at 14°C, by measuring the change in the absorbance at 600 nm using a microplate reader (BioTek). The growth inhibition curves were generated by plotting the OD at 600 nm and the peptide concentration. The MIC was considered as the lowest concentration of PACAP at which no bacterial growth was detected (an OD_600_ of 0).

### RTS11 Exposure Trials

#### Exposure to PACAP

In 6-well tissue culture plates (ThermoFisher), RTS11 was seeded at 1.5 x 10^6^ cells/well in 1.5 mL of L-15 media with no antibiotics and maintained overnight at 14°C. Cells were exposed to PACAP concentrations of either 0.0002 μM, 0.002 μM, 0.02 μM, 0.1 μM, 0.2 μM, 2 μM, 20 μM, or a no PACAP control to a final volume of 4 mL per well. Following this single exposure to PACAP, all experimental plates were returned to the 14°C incubator. On days 1, 2, and 3, the supernatant was collected from experimental wells and adherent cells were mechanically dislodged using a sterile 23 cm cell scraper (ThermoFisher) and added to the supernatant of respective wells. All wells were then washed with 1 mL of phosphate buffered saline (PBS, Gibco) which was also added to the appropriate supernatant/cell mixture. The cells were centrifuged (5 min, 500 × g, 4°C), washed once with 5 mL of PBS, and the resulting cell pellets were stored at −80°C for future use.

#### Simultaneous Exposure to Both PACAP and Live *F. psychrophilum*

In a second experiment, RTS11 was exposed to PACAP concentrations of 0.0002 μM, 0.002 μM, 0.02 μM, and 0.1 μM in similar conditions as described above in the first PACAP trial (section Exposure to PACAP). Prior to the single addition of PACAP, 0.5 mL of FPG101 was added to each well at bacterial concentrations ranging from 1.3–2.0 × 10^6^ CFU/mL (multiplicity of infection [MOI] of 0.7–1.3). Sampling was completed as described above (section Exposure to PACAP).

#### Pre-treatment With PACAP Followed by Infection With Live *F. psychrophilum*

It was observed that *F. psychrophilum* grew rapidly in wells when exposed to RTS11 simultaneously with PACAP. Because it was possible that PACAP might not be able to influence either RTS11 alone, the *F. psychrophilum* alone, or both due to this rapid growth, PACAP was added to RTS11 wells 24 h before the addition of live *F. psychrophilum*. Otherwise all procedures for sample collection and exposure were identical to those described above in the first PACAP experiment (section Exposure to PACAP).

### Survival of RTS11 Following PACAP Exposure

To determine whether PACAP negatively influenced RTS11 viability, the cells were exposed to a single dose of 0.002 μM, 0.02 μM, 0.1 μM, 0.2 μM, 2 μM, 20 μM, or a no PACAP control as described above. On days 1, 2, and 3 following this exposure, the supernatant was collected from experimental wells and any adherent cells were detached using 400 μL of 0.25% trypsin-EDTA (Gibco) and the wells washed with 1 mL of PBS. These trypsinized cells were combined with the collected supernatant which was then centrifuged 500 × g for 5 min at 4°C. The cell pellet was washed twice with 1 mL of PBS before resuspending in 200 μL of PBS. To determine RTS11 cell viability after exposure to PACAP, a trypan blue (Sigma) exclusion test was performed using a haemocytometer under a phase contrast microscope (Leica). This experiment was repeated three times.

### Presence of Viable *F. psychrophilum* in RTS11 Cell Cultures Following PACAP Exposure

In six 6-well plates, quadruple wells of RTS11 cells were exposed to either PACAP and *F. psychrophilum* simultaneously or to 24 h pre-treatment of PACAP prior to the addition of *F. psychrophilum* as described above. All experiments had a MOI of 1. On days 2 and 3 post-infection with live *F. psychrophilum*, 500 μL of the supernatant from each well was removed and serially diluted for an SPC assay to determine the number of viable bacterial cells in the supernatant. Otherwise the RTS11 cells for each day were collected as described above and pellets were frozen at −80°C for future RNA extraction.

### Permeabilization Assay

FPG101 was grown as described above for the MIC assay in section Minimum Inhibitory Concentration (MIC) of *C. gariepinus* PACAP-38 on *F. pyschrophilum*. One milliliter of the final bacterial culture was removed and boiled for 20 min to act as a heat-killed control. After boiling, 100 μL of the heat-killed FPG101 was spread onto a CA plate to confirm the absence of viable bacteria.

In a sterile, 96-well BioLite plate (ThermoFisher), 90 μL of live bacterial culture was added to all experimental wells. In triplicate, 10 μL of either PACAP or the synthetic HSP70 peptide fragment to reach final concentrations of 50 μM PACAP, 30 μM PACAP, 0.1 μM PACAP, and 50 μM HSP70. As a live bacteria control, 10 μL of cytophaga broth alone was added to triplicate wells of the live FPG101 culture. As a negative control, 90 μL of heat-killed FPG101 was added to triplicate wells and filled to 100 μL with CB. As a blank, triplicate wells received 100 μL of CB. The assay plate received gentle shaking to mix well contents and was incubated at 14°C for 72 h. Following incubation, each well received 100 μL of 2X BacLight solution (ThermoFisher, L13152) and was incubated in the dark for 15 min. Because the BacLight solution consists of both SYTO 9 (6 μM) and propidium iodide (30 μM), the plate was read at an excitation of 485 nm and an emission of 530 nm for SYTO 9 (green) as well as an excitation of 485 nm and an emission of 630 nm for propidium iodide (red). The reads were completed using a Synergy H1 plate reader (BioTek Instruments). The bacterial fluorescent intensities (F_cell_) were calculated as a ratio of F_cell_530/F_cell_630 and presented as the green/red fluorescence ratio.

### qRT-PCR

#### RNA Extraction and cDNA Synthesis

RNA was extracted from RTS11 cell pellets (1.5 × 10^6^ cells) using an RNeasy RNA Extraction Kit (Qiagen) as described by the manufacturer. To remove any contaminating genomic DNA, all RNA samples were treated with DNase I (Thermo Scientific). RNA samples were then quantified using the Take3 plate of a Synergy H1 plate reader (BioTek Instruments) and were stored at −80°C until further use. Complementary DNA (cDNA) was synthesized from 500 ng of total RNA using the qScript cDNA Supermix (Quanta Biosciences) in accordance to the manufacturer's instructions. For a no template control, 500 ng of RNA suspended in 20 uL of DEPC water was included in the cDNA synthesis reaction without reverse transcriptase.

#### qRT-PCR Reactions

To assess transcript levels of *IL-1*β, *TNF*α, *IL-6, PAC1*, and *VPAC1* in RTS11 cells, qRT-PCR analysis was completed. All PCR reactions were 10 μl and contained: 2.5 μl of cDNA (25 ng/μl diluted 1:10 in RNase free water), 2x WISENT ADVANCED™ qPCR mastermix (Wisent), and forward and reverse primers (Sigma Aldrich) to a final working concentration of 0.25 μM. All qPCR reactions were completed on the LightCycler® 480 II (Roche). The sequences for all primer sets are outlined in Table 1. Each experimental sample was run in triplicate. For each plate, triplicate wells of a calibrator, no template control and RNA only control were also present. The program used for all qRT-PCR reactions was as follows: pre-incubation at 95°C for 10 min followed by 40 cycles of denaturation at 95°C for 10 sec, annealing at 60°C for 5 s and extension at 72°C for 8 s. A melting curve was completed for every run from 65 to 97°C with a read every 5 s. Product specificity was determined through single PCR melting peaks. All qRT-PCR data was analyzed using the ΔΔCt method and is presented as the average of 3 experimental replicates with the standard deviation. Specifically, gene expression was normalized to the reference gene (EF1α) and expressed as fold change over the day 0 control group where control expression was set to 1.

**Table 1:** Primers used for qRT-PCR analysis of RTS11.

**Primer name**	**Sequence (5^**′**^-3^**′**^)**	**References**
IL-1β	**F:** CCACAAAGTGCATTTGAAC	(54)
	**R:** GCAACCTCCTCTAGGTGC	
TNFα	**F:** GTGCAAAAGATACCCACC	(54)
	**R:** CACTGCACGGTGTCAG	
IL-6	**F:** CTTCTACACGCTATCTCTCACTC	(54)
	**R:** CGTCTGTCCCGAGCT	
VPAC1	**F:** CAGGTGAAAATTGGTTACACTGTTG	(55)
	**R:** TAGTTCCTAGTGCAGTGGAGTTTCC	
PAC1	**F:** TGAACCTGTTTGTGTCATTCATTCT	(55)
	**R:** ACACTCCACAGTGTGTAAGAAGCAG	
EF1α	**F:** CGCACAGTAACACCGAAACTAATTAAGC	(54)
	**R:** GCCTCCGCACTTGTAGATCAGATG	

### Statistics

All statistical analyses were completed using the statistical software Statistica version 7 (StatSoft, Tulsa, OK). Prior to the completion of the appropriate statistical test, a normal distribution and equal variance was confirmed. A one-way ANOVA was completed for the growth of *F. psychrophilum* in RTS11 cultures and the permeabilization assay. Whereas, a two-way ANOVA was completed for all qRT-PCR results and analyzing the viability of RTS11 to various PACAP concentrations. The appropriate ANOVA test was then followed by a Fisher's least significant difference (LSD) *post-hoc* test to determine significant differences.

## Results

### Minimum Inhibitory Concentration (MIC)

**Figure 1:**
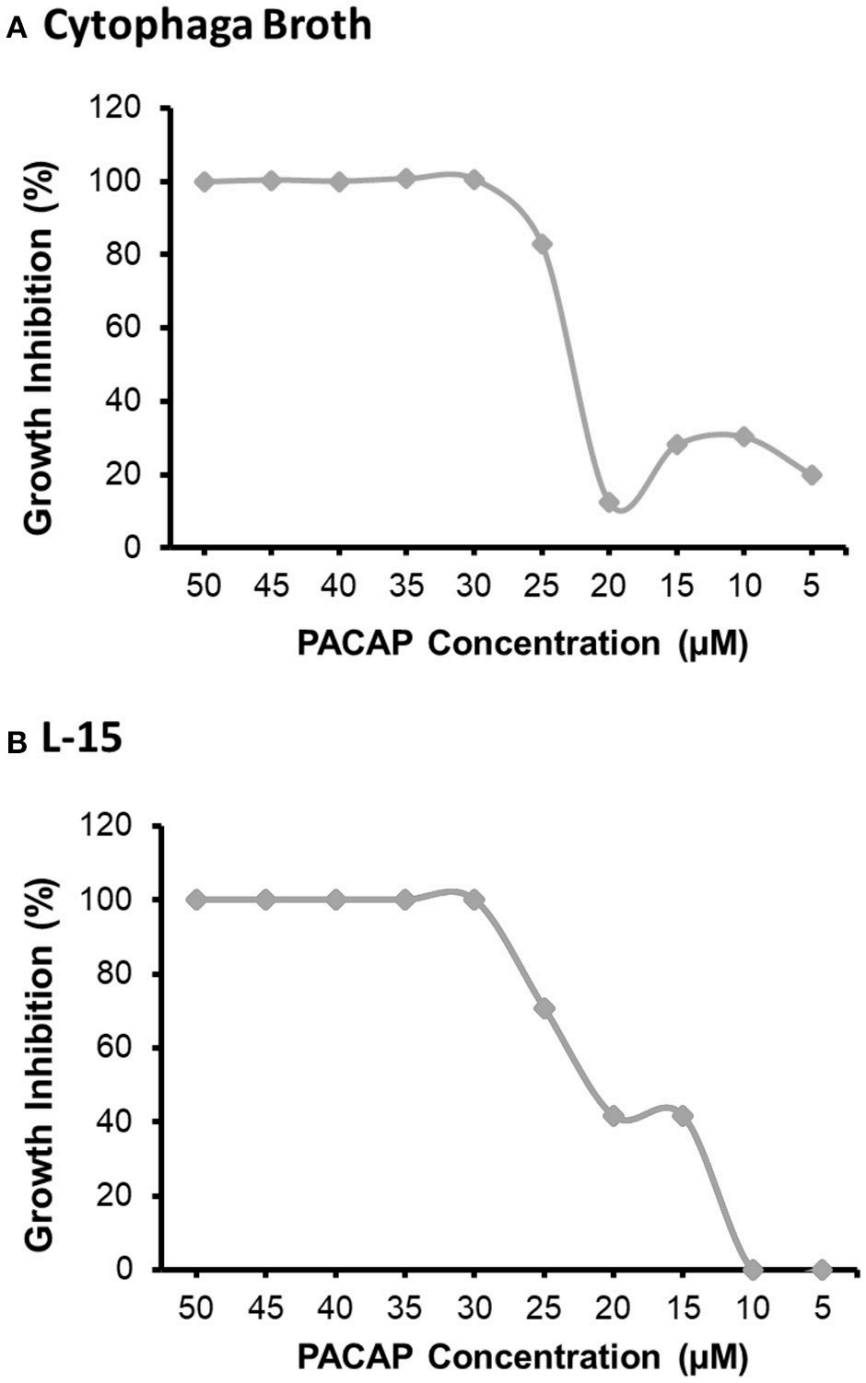
The minimum inhibitory concentration (MIC) of *C. gariepinus* PACAP-38 required to prevent the growth of *F. psychrophilum* alone. The MIC was determined following 3 days of growth in both cytophaga broth **(A)** and in Leibovitz-15 (L-15) cell culture media **(B)**. Each panel represents the results of three independent experiments.

The MIC was analyzed using both the preferred growth medium of *F. psychrophilum*, cytophaga broth (CB), and the L-15 cell culture media used to sustain the RTS11 cultures (Figure 1). For both CB (Figure 1A) and L-15 (Figure 1B), the MIC was found to be 30 μM. It appears that PACAP can maintain its antimicrobial function in both media types, thus the function of this peptide could be assessed during *in vitro* live infection experiments with RTS11.

### Impact of PACAP Concentrations on RTS11 Survival

**Figure 2:**
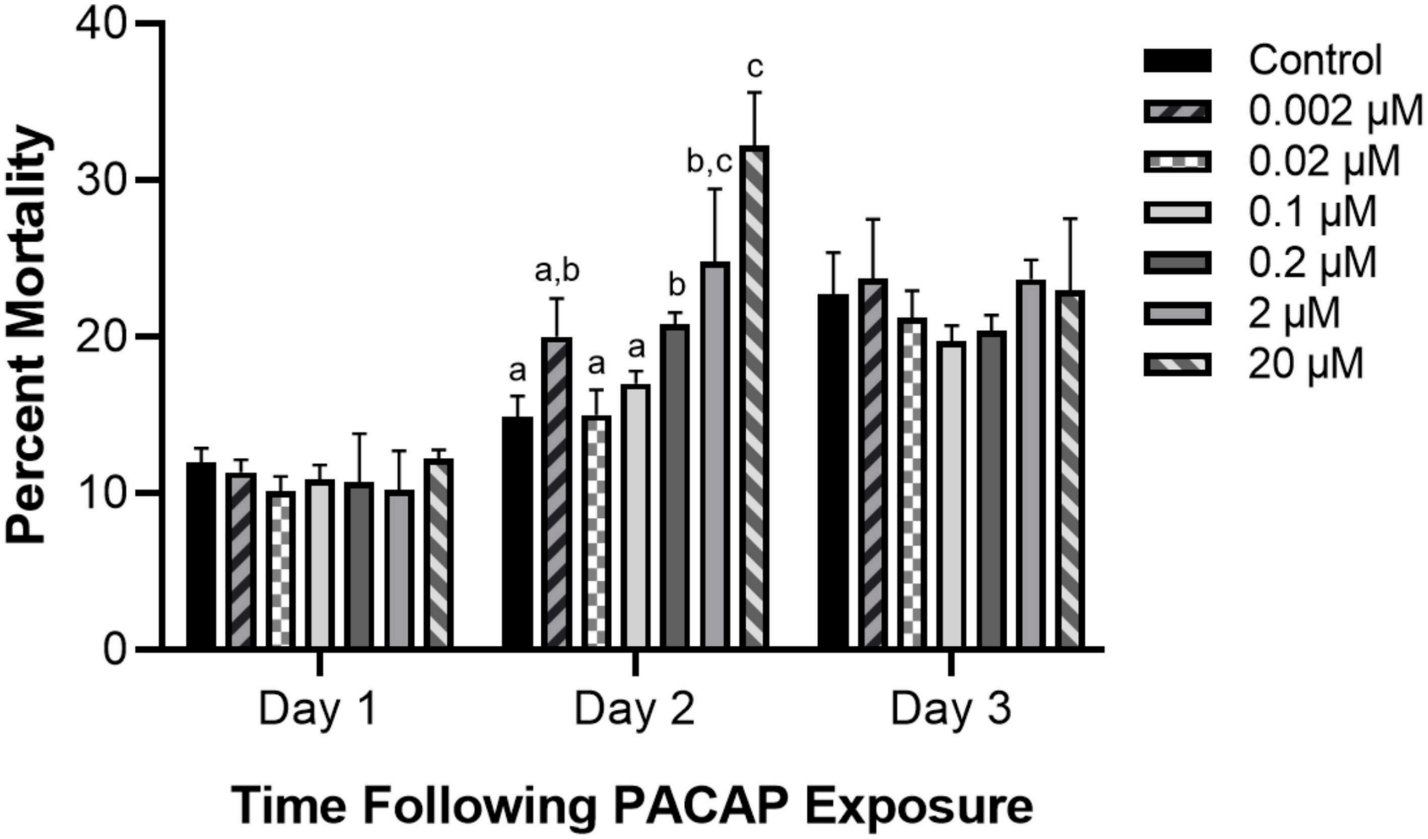
Impact of *C. gariepinus* PACAP-38 on RTS11 viability. RTS11 was exposed to varying concentrations of PACAP ranging from 0–20 μM and the percent mortality of RTS11 was determined for each concentration using a trypan blue assay. This figure represents three independent experiments where a *p* < 0.05 was considered to be significantly different when compared to the no PACAP control for individual timepoints. All vertical error bars represent the standard deviation (SD). Lowercase letters denote significant differences at *p* < 0.05.

Even if PACAP is capable of killing aquatic bacterial pathogens, this ability has reduced value if the peptide negatively impacts the survival of rainbow trout immune cells as well. Based on the six concentrations of PACAP analyzed here (ranging from 0.002–20 μM), only PACAP concentrations of 0.2 μM and higher significantly decreased the viability of RTS11 (Figure 2). Furthermore, cell death was only observed on day 2 of exposure. Cell viability was not significantly different between PACAP concentrations on both days 1 and 3 of exposure.

### Effect of PACAP on *F. psychrophilum* Growth Throughout RTS11 Infections

**Figure 3:**
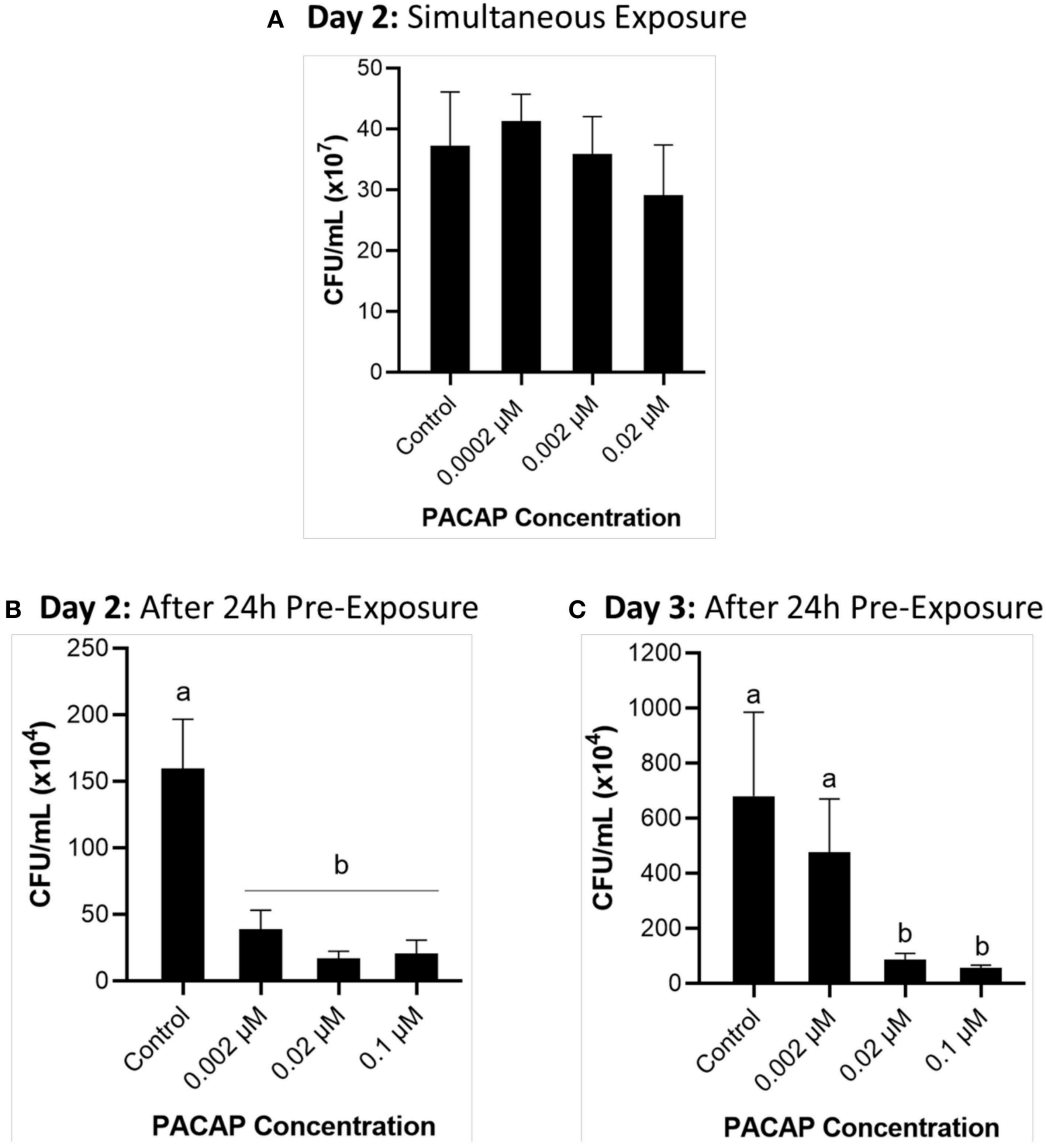
Quantification of *F. psychrophilum* by standard plate count (SPC) of cell culture media during live infection (MOI of 0.7-1.3) of PACAP-treated RTS11. RTS11 was exposed to live *F. psychrophilum* either alone or in combination with *C. gariepinus* PACAP-38 concentrations (0.0002–0.02 μM) and CFU/mL was calculated on day 3 **(A)**. RTS11 pre-treated with PACAP-38 concentrations (0.002–0.1 μM) 24 h before exposure to live *F. psychrophilum* and the CFU/mL was calculated on day 2 post-infection **(B)** and day 3 post-infection **(C)**. Each panel represents the results of three independent experiments where a *p* < 0.05 was considered to be significantly different when compared to the no PACAP control (i.e., RTS11 exposed to only live *F. psychrophilum*). All vertical error bars represent the SD. Lowercase letters denote significant differences at *p* < 0.05.

When RTS11 was exposed to live *F. psychrophilum* simultaneously with various PACAP concentrations, the number of viable bacteria present in the supernatant was not significantly different when compared to that of the no PACAP control on day 2 (Figure 3A). As it was possible that PACAP required time to stimulate a defensive immune state in RTS11, this experiment was repeated but this time the cells were exposed to PACAP concentrations 24 h prior to receiving the infectious dose of *F. psychrophilum*. When using this experimental design, all three concentrations of PACAP (0.002, 0.02, and 0.1 μM) were shown to significantly reduce the number of viable bacteria in the RTS11 on day 2 (Figure 3B). This reduction was still observed on day 3 but was only found to be significant in the two higher concentrations of PACAP at 0.02 and 0.1 μM (Figure 3C).

### Permeabilization of *F. psychrophilum* by PACAP

**Figure 4:**
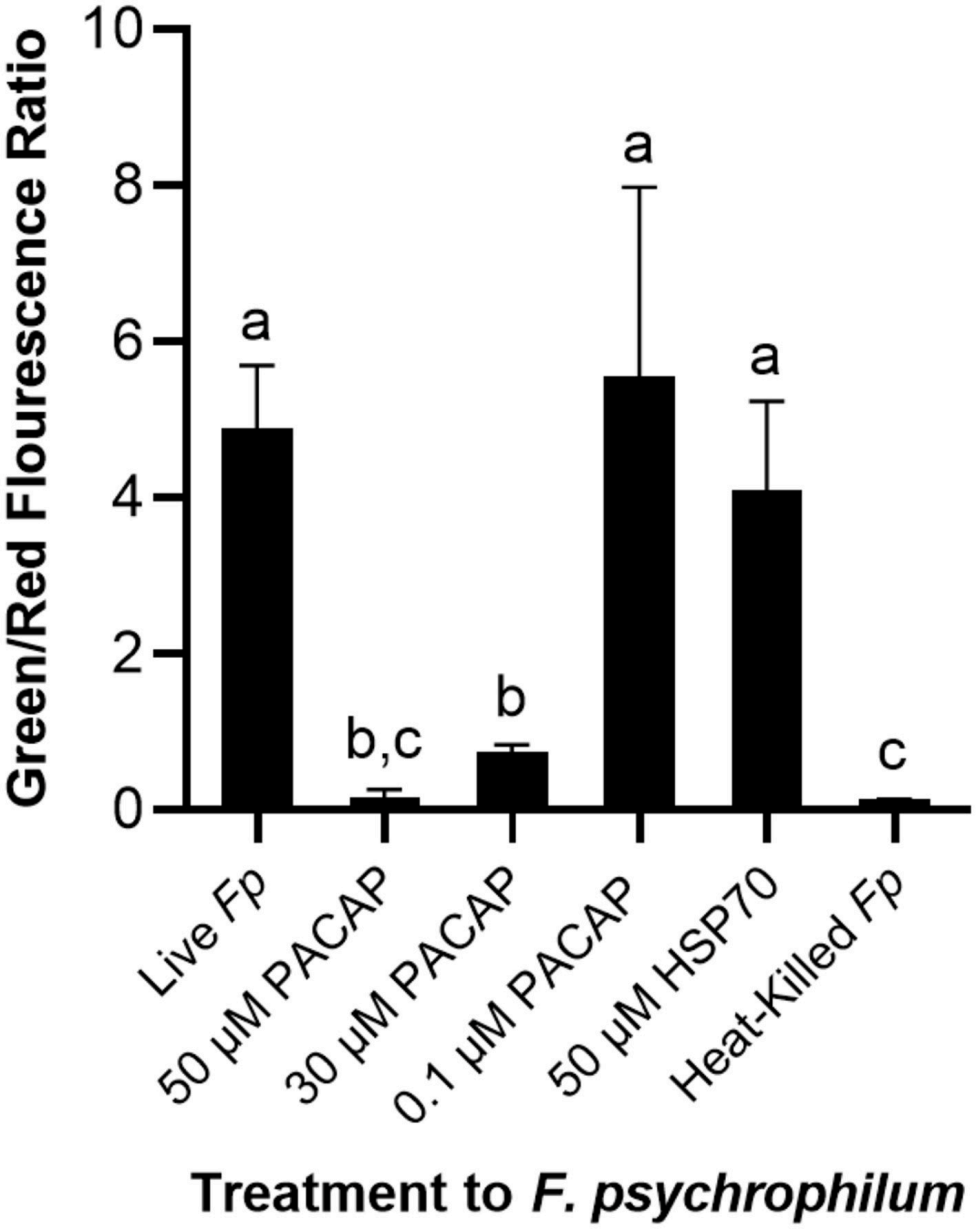
Disruption of *F. psychrophilum* membrane by PACAP-38 of *C. gariepinus*. Live *F. psychrophilum* was grown alone, in the presence of PACAP-38 (50, 30, and 0.1 μM) and in the presence of a control synthetic peptide fragment of comparable size, 50 μM HSP70 (1.3 kDa). As a negative control, heat-killed *F. psychrophilum* was also included. Following a 3 day incubation at 14°C, all experimental wells were exposed to BacLight which would cause live bacterial cells to fluoresce green (SYTO9) and permeabilized cells to fluoresce red (propidium iodide). The ratio of green/red fluorescence was calculated, and this value was compared between conditions. This experiment was replicated four times and the averages are presented as the means + SD. A *p* < 0.05 was considered to be statistically significant. Lowercase letters denote significant differences at *p* < 0.05.

To establish whether the studied PACAP concentrations were either inducing direct lysis of *F. psychrophilum* or instead stimulating RTS11 to respond to and destroy the bacterial pathogen, a permeabilization assay was performed. At doses comparable to the MIC (50 and 30 μM), PACAP was shown to induce permeabilization of *F. psychrophilum* comparable to that observed when the bacterium was heat-killed (Figure 4). Interestingly, this ability was absent when using 0.1 μM of PACAP as, in this case, the bacteria presented reduced permeabilization similar to that of the live *F. psychrophilum* control (Figure 4). The permeabilization ability noted here was also specific to PACAP as 50 μM of a synthetic peptide fragment of comparable size (1.3 kDa), HSP70, was not able to permeabilize *F. psychrophilum*.

### Influence of *C. gariepinus* PACAP-38 on RTS11 Immune Gene Expression

#### Exposure to PACAP

**Figure 5:**
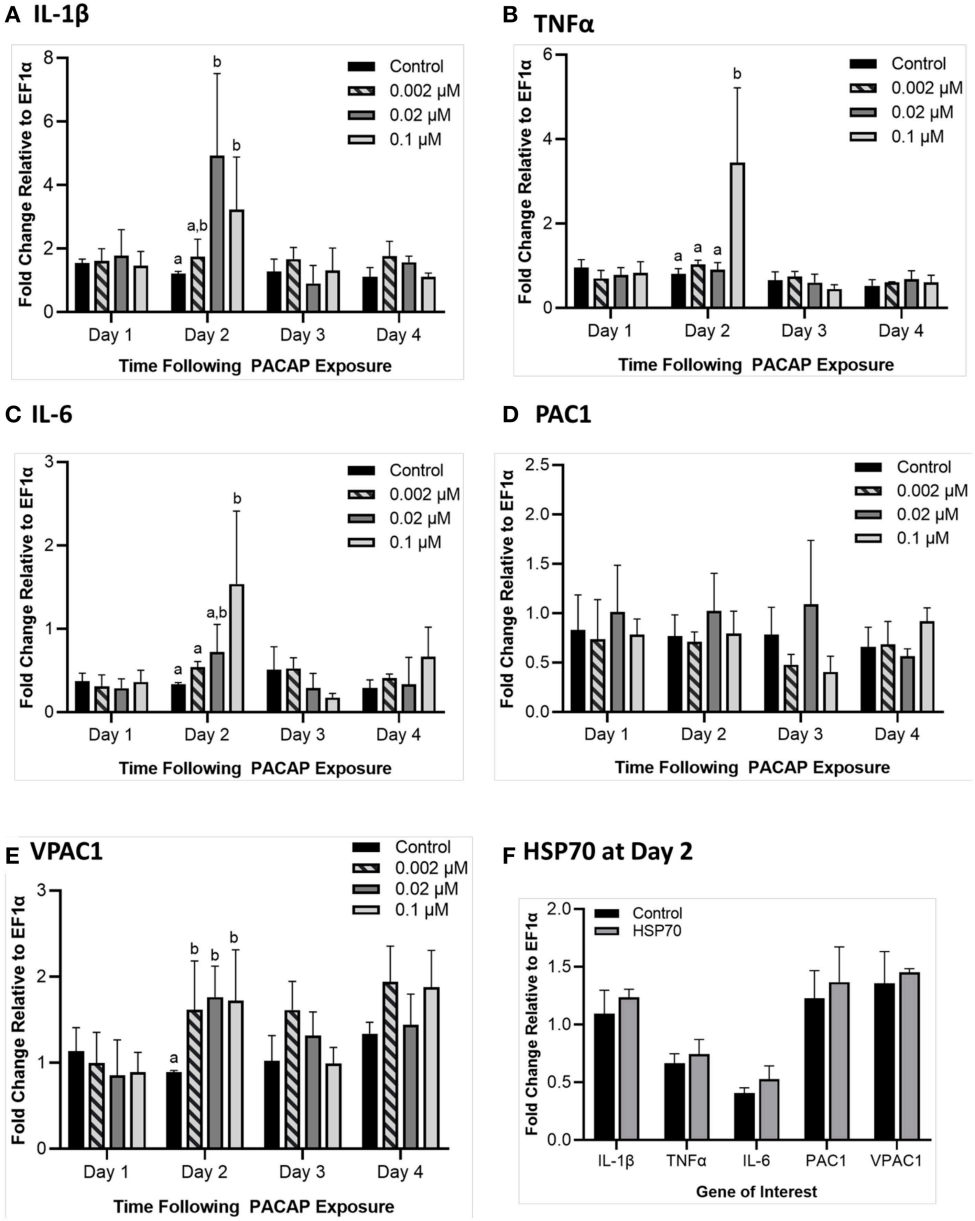
Impact of *C. gariepinus* PACAP-38 (0.002 μM, 0.02 μM and 0.1 μM) on RTS11 pro-inflammatory cytokines and receptors PAC1 and VPAC1 mRNA expression 1–4 days following peptide exposure. Transcript expression of pro-inflammatory cytokines *IL-1*β **(A)**, *TNF*α **(B)** and *IL-6* **(C)** was measured so that the influence of PACAP alone on immune function could be assessed. The present PACAP receptors *PAC1* **(D)** and *VPAC1* **(E)** were also measured to determine whether PACAP could prime RTS11 cells to bind more PACAP. Because 1 day after the pre-exposure appeared to be the only timepoint with significant upregulation due to PACAP, RTS11 was also exposed to 0.1 μM of a synthetic HSP70 peptide fragment control of comparable size (1.3 kDa), for 48 h to confirm that this stimulation was due to PACAP and not a property of synthetic peptides alone **(F)**. All panels represent three independent experiments and are presented as means + SD. A *p* < 0.05 was considered to be statistically significant when compared to the no PACAP control for each timepoint. Lowercase letters denote significant differences at *p* < 0.05.

To determine whether PACAP alone could stimulate a response in RTS11, the cells were exposed to various concentrations of the peptide (0.002, 0.02, and 0.1 μM) over 4 days. Following this exposure, gene expression of pro-inflammatory cytokines (*IL-1*β, *TNF*α, and *IL-6*) and PACAP receptors (*PAC1* and *VPAC1*) were measured using qRT-PCR. For all three of the pro-inflammatory cytokines measured, a significant difference was only seen on day 2 (Figures 5A–C). Furthermore, for *TNF*α and *IL-6*, this significant increase was only observed at the highest PACAP concentration of 0.1 μM. Meanwhile for *IL-1*β, a significant increase occurred at both 0.02 μM and 0.1 μM of PACAP. When regarding the PACAP receptors, there were no significant increases observed for *PAC1* but by day 2, *VPAC1* significantly increased at all of the concentrations studied (Figures 5D–E). To confirm that this response was specific to PACAP and not just a property of synthetic peptides in general, RTS11 was also exposed to 0.1 μM of a synthetic peptide fragment of rainbow trout HSP70, which was unable to induce significant expression differences in all of the genes selected for this study (Figure 5F).

#### Exposure to PACAP 24 h Before *F. psychrophilum* Infection

**Figure 6:**
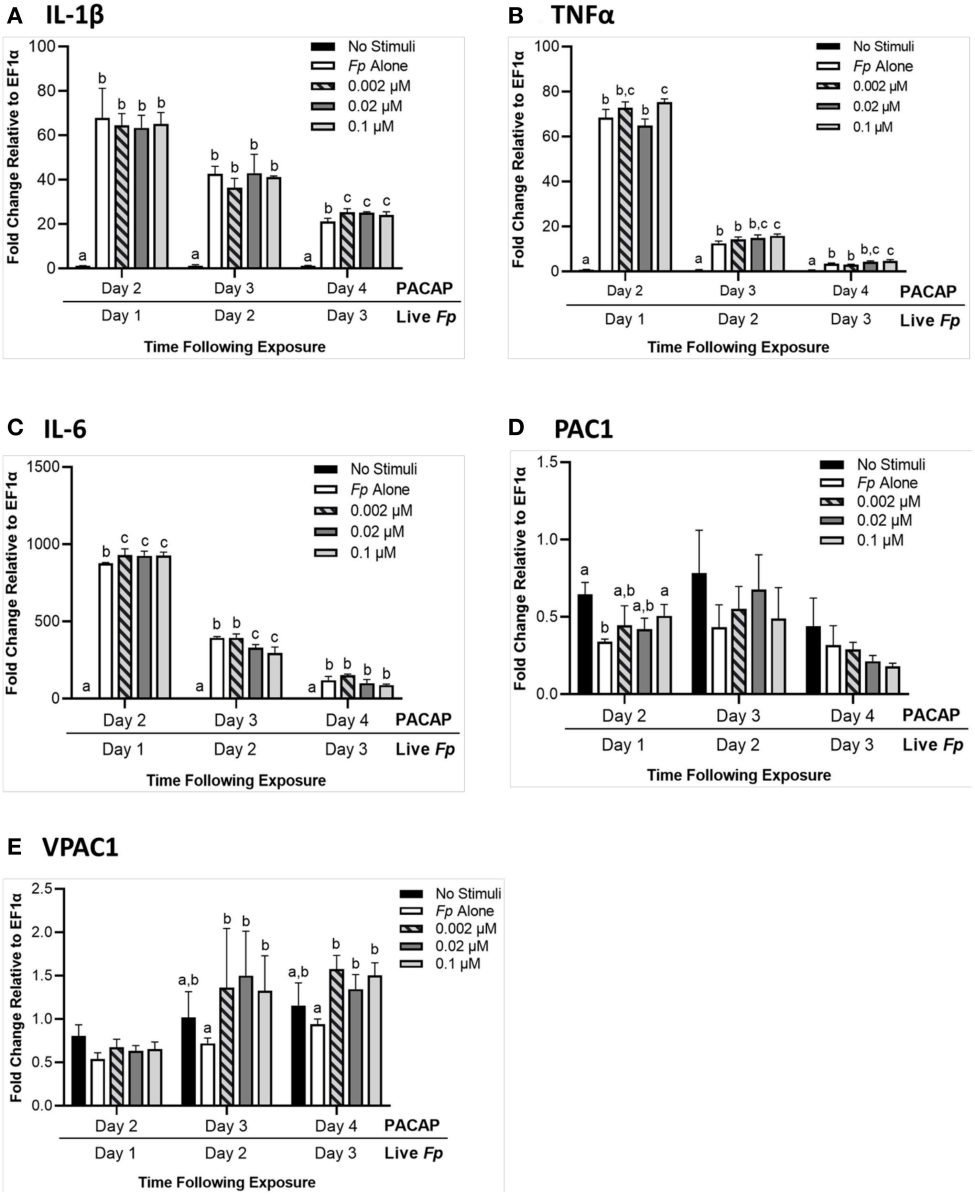
RTS11 transcript expression when challenged with live *F. psychrophilum* following 24 h pre-treatment with PACAP concentrations (0.002, 0.02, and 0.1 μM). RTS11 that was not exposed to PACAP or live *F. psychrophilum* was also included as a no stimuli control. To determine whether PACAP has immunomodulatory effects during live infection, transcripts of the pro-inflammatory cytokines *IL-1*β **(A)**, *TNF*α **(B)** and *IL-6* **(C)** were measured in RTS11 on days 1, 2, and 3 of infection. The PACAP receptors that are found within RTS11, *PAC1* **(D)** and *VPAC1* **(E)** were also assessed throughout the live infection challenge. All panels represent three independent experiments and are presented as means + SD. A *p* < 0.05 was considered to be statistically significant when samples were compared within each timepoint. Lowercase letters denote significant differences at *p* < 0.05.

When RTS11 was challenged with live *F. psychrophilum* infection 24 h after exposure to PACAP, there were some interesting differences in transcript expression that were not observed during PACAP exposure alone (Figure 5). For *IL-1*β expression, there were no significant differences at 1 and 2 days post-infection when compared to the RTS11 cells exposed to live pathogen alone. However, by day 3 of infection, a significant increase in *IL-1*β transcripts was observed for all three concentrations of PACAP (Figure 6A). In comparison, *TNF*α expression was significantly upregulated at day 1, 2, and 3 post-infection but only at 0.1 μM of PACAP, the highest concentration of the AMP (Figure 6B). Interestingly, all three concentrations of PACAP showed a significant increase in *IL-6* expression on day 1 post-infection but by day 2 this upregulation was either lost at 0.002 μM or was significantly reduced in the two higher concentrations of PACAP (Figure 6C). When compared to the PACAP only expression (Figure 5), the PACAP receptors were also influenced differently during pathogen challenge. On day 1 post-infection, *PAC1* showed a significant increase only 0.1 μM (Figure 6D). Meanwhile, *VPAC1* showed a significant increase at day 2 in the two higher PACAP concentrations (0.02 and 0.1 μM) and on day 3, all three concentrations of PACAP presented a significant upregulation when compared to the control cells that were exposed to *F. psychrophilum* alone (Figure 6E).

## Discussion

### Influence of PACAP on the Viability of *F. psychrophilum* and RTS11 *in vitro*

Currently aquaculture facilities have very few options outside of antibiotics to combat disease outbreaks. When this is combined with the rising incidence of multi-drug resistance, AMPs such as PACAP are promising alternatives for disease control/prevention. The purpose of this study was to evaluate the antimicrobial activity and immunomodulatory function of PACAP within a live infection model consisting of RTS11 and the coldwater pathogen, *F. psychrophilum*. To assess the efficacy of this proposed system, it was critical to determine the impact that PACAP alone had on both components of the infection model: the host and the bacterium. For several bacterial pathogens, PACAP has been shown to have a direct antimicrobial effect (29, 46) including those of aquatic origin (46). Thus, it was not surprising that PACAP presented a similar result when *F. psychrophilum* was exposed to various concentrations in a preferred growth medium, cytophaga broth. Additionally, the MIC of PACAP was not influenced when *F. psychrophilum* was grown in cell culture media, a substance meant to mimic physiological conditions [reviewed by Yao and Asayama (56)]. This suggests that synthetic PACAP may maintain its antimicrobial effects in some physiological conditions, as may be the case when administered to live organisms. But despite this promising observation, it is important to remember that disease outbreaks in aquaculture settings would differ significantly from microbial culture settings in important ways. Namely the assumption of sterility and the resulting absence of competing microorganisms. As such, concentrations surrounding the observed MIC may not represent an effective therapeutic dose to control/prevent live infection and prove to be suboptimal for the host organism if not properly evaluated.

Aside from their potential as antimicrobials and immunomodulators, AMPs are also praised for potentially having minimal negative effects on mammalian host cells (57) including those of immune origin (58). Quite often this “cell selectivity” is based on the concentration required for the AMP to induce 50% hemolysis in host red blood cells (RBCs). If this concentration is much higher than what is required for the MIC, the peptide is considered to be essentially non-toxic to host cells [reviewed by Matsuzaki (59)]. This has been shown with *C. gariepinus* PACAP-38 when both human and fish RBCs were exposed to the peptide, revealing only RBC lysis at extremely high concentrations (46). Unfortunately, these methods of measurement are not always directly comparable as antimicrobial assays generally use a bacterial concentration of ~5 × 10^5^ CFU/mL while hemolysis assays use what corresponds to be 6 × 10^8^ cells/mL [reviewed by Matsuzaki (59)]. In fact, when Imura et al. (60) corrected for this concentration difference during their analyses of the antimicrobial peptide magainin, the MIC concentration of 10 μM was enough to completely lyse the host RBCs (60). In the present study, RTS11 was exposed to PACAP wherein higher concentrations (0.2, 2, and 20 μM) had a significantly negative impact on RTS11 viability. Some studies have alluded to the idea that AMPs may be toxic to mammalian cells in the absence of microorganisms (60), thus it is possible that this may also be observed in fish cells. There are important differences between prokaryotic and eukaryotic membranes that may improve the chance that AMPs will preferentially bind to the membrane of microorganisms before host cells (reviewed by 58). Prokaryotic membranes have a high negative charge due to being predominantly composed of phosphatidylglycerol, cardiolipin, or phosphatidylserine, thus have a greater chance of attracting the cationic peptides that are AMPs. In comparison, mammalian cells may be less attractive for AMP penetration as they are enriched in zwitterionic phospholipids resulting in an overall neutral charge [reviewed by Matsuzaki (61) and Huang et al. (62)]. Furthermore, mammalian cell membranes contain cholesterol, something that is absent in prokaryotic membranes. Interestingly, it has been shown that cholesterol can dramatically reduce the activity of AMPs (63) providing another potential layer of protection for mammalian cells. But despite these important differences that may help with membrane selection of AMPs, complete protection of the host cells from AMP-induced cytotoxicity may not be possible at higher AMP concentrations. To even consider PACAP for use as a therapeutic agent in aquaculture settings, the therapeutic dose must not be cytotoxic to the host, whether infected or microbe-free.

### Assessing the Ability of PACAP to Inhibit Bacterial Growth During Live Infection of RTS11 Cells

In spite of the value that can be obtained from determining the MIC of PACAP in various culture media, as well as the ideal concentration for survival of host cells, the observed antimicrobial activity is meaningless if it is lost or not effective during live pathogen challenge.

The current study is the first of its kind that has demonstrated the antimicrobial activity of PACAP during an *in vitro* live infection model with an aquatic pathogen. But interestingly, the teleostean version of PACAP was only able to reduce the viable bacterial count of *F. psychrophilum* when RTS11 cells were pre-treated with PACAP for 24 h. It appears that rainbow trout macrophages require time to respond and activate an effective immune response when exposed to *F. psychrophilum*. As obligate poikilotherms, metabolic rates in fish are heavily influenced by their environmental temperature (64, 65). Because cells derived from rainbow trout, a coldwater salmonid, are grown at much lower temperatures than their mammalian counterparts, it may take more time for these cells to respond to stimuli. Indeed this has been shown with both RTS11 and rainbow trout B cells where the chemoattractant ability of the chemokine CK9 strongly increased when the cells were pre-treated with T-independent antigen (66). Likewise, in rainbow trout primary head kidney culture, the cells sometimes required 48 h before an increase in respiratory burst activity was observed (67). In both of these examples, the immune cells were maintained at 18°C, but in the current study, RTS11 was held at 14°C as this is a relevant temperature at which BCWD occurs (68). Thus, a longer pre-treatment time at this lower temperature may be required depending on the response that is being measured. Though a temperature between 8 and 14°C would be optimal for testing the efficacy of PACAP in protecting rainbow trout from infection with *F. psychrophilum*, there are many infectious diseases that influence the culture of numerous aquatic organisms. As a result, experimental doses with therapeutic AMPs must be tested *in vivo* to ensure that they will provide protection and effective immune stimulation toward relevant pathogens within an applicable temperature range.

Cell culture systems provide a controlled, cost-effective method for exploring numerous biological phenomena, but it is important to recognize the limits of these models. In a cell culture setting, individual cells are directly exposed to the experimental stimulant, without physiological barriers or a complicated cellular milieu to overcome. As a result, *in vitro* systems often have much lower doses than what is appropriate within the whole organism. This was displayed when Gotlieb et al. (69) used several methods to isolate and stimulate NK cells revealing that the cells were 10–30 times more susceptible to stress hormones *in vitro* than what was observed when stimulated in plasma, a much more biologically relevant medium. Though the effects of PACAP on fish infections *in vivo* have not yet been explored, there have been several studies to determine the impact of this AMP on growth, immunomodulation and physiology (48, 70–72). One study by Lugo et al. (48) exposed juvenile fish to an average of 4 μg of PACAP per fish, which significantly enhanced tilapia growth. In comparison, with the RTS11 infection model presented here, each well received 1.8 μg or less and antimicrobial activity was still observed. Though the PACAP doses optimized for the current *in vitro* study were very effective, an *in vivo* model would require further optimization to develop an efficacious exposure range.

### Confirming the Direct Mode of Action of PACAP on *F. psychrophilum*

When it comes to bacterial pathogens, the consensus regarding AMP function is that they are either membrane disrupting, or non-membrane disrupting [reviewed by Bahar and Ren (9)]. Though PACAP was able to lower the number of viable bacteria when grown alone in media (for the MIC) and in RTS11 cultures throughout live infection, it was unclear whether this was due to the direct antimicrobial activity of PACAP acting on *F. psychrophilum*. Previous work with mammalian PACAP confirmed for the first time that the peptide was capable of disrupting membranes of relevant terrestrial bacterial pathogens (29). This permeabilization is a common mode of action for α-helical AMPs (73), including PACAP, but until now this ability has not been confirmed for the version of PACAP produced by fish. Previous bioinformatic analysis of PACAP-38 from *C. garipinus* provided evidence that this peptide was very likely to have cell penetrating properties (46). The present study was able to functionally validate the ability of *C. gariepinus* PACAP-38 to permeabilize the membrane of *F. psychrophilum* at concentrations surrounding the MIC. Furthermore, this was an ability specific to PACAP as another synthetic peptide fragment of comparable size from a teleost, HSP70, did not induce permeabilization. Interestingly, at the highest concentration that reduced the viable bacterial count during live infection, 0.1 μM, PACAP was unable to permeabilize the membrane of *F. psychrophilum*. This finding confirmed that at 0.1 μM, one of the many other effects that PACAP may have on teleostean immunity must have been responsible for stimulating RTS11 to destroy and/or slow the growth of the coldwater pathogen.

### Understanding the Immunostimulatory Effects of PACAP on RTS11

PACAP has been shown to have immunomodulatory effects on whole fish and in fish cells (46, 70, 71, 74) but this has only been studied in the absence of live infection. Furthermore, there has been limited research regarding the activity of PACAP directly on teleost immune cells. Specifically with RTS11, the immunostimulatory effect of three other α-helical AMPs has been reported (75) but not when dealing with a live bacterial challenge. The present study explores, for the first time, the impact of relevant doses of PACAP on the immune function of RTS11 in both the presence and absence of *F. psychrophilum*. In the absence of bacterial infection, PACAP was shown to stimulate pro-inflammatory cytokine expression 48 h following PACAP treatment as well increasing the expression of one of the PACAP receptors, *VPAC1*. When analyzing PACAP receptor expression in RTS11, it is important to note that only *VPAC1* and *PAC1* receptor genes were measured as it has previously been shown by Lugo et al. (55) that these cells do not express the third receptor gene, *VPAC2*. Lugo et al. (55) also showed that despite *PAC1* presenting the highest constitutive expression in all rainbow trout lymphoid tissues *in vivo*, this was not observed in RTS11 where *VPAC1* presents the greatest expression. As *PAC1* has been found to be a fundamental type I receptor for PACAP this result was unexpected. Nonetheless, the current study validated this finding when *VPAC1* presented significant upregulation following RTS11 stimulation with PACAP while *PAC1* did not. As PACAP was shown to stimulate a slight increase in pro-inflammatory cytokines and upregulate the expression of *VPAC1*, pre-treatment with this AMP may stimulate a protective state within the rainbow trout immune cells.

In itself, the presence of live bacteria would be capable of inducing pro-inflammatory cytokine expression in RTS11. But when this was combined with 24-h pre-treatments with PACAP, infection with *F. psychrophilum* had an effect on transcript expression that was quite different from PACAP alone. Rather than all three of the studied pro-inflammatory cytokines increasing their expression at the same time, each one showed significant upregulation at different time points post-infection when compared to RTS11 exposed to the bacteria alone. The pattern of enhancing inflammatory cytokine production following pre-treatment is similar to that of trained immunity that has been observed in mammalian monocytes and macrophages (76). Quintin et al. (76) showed that when primed with β-glucan prior to exposure to LPS, monocytes and macrophages were able to induce a greater pro-inflammatory response than unprimed cells. Perhaps PACAP has a similar function and is able to prime immune cells to produce a faster, more damaging response when they come into contact with a live pathogen. This would provide an explanation for the observed decrease in viable bacteria following PACAP exposure at doses that were not able to directly permeabilize the membrane of *F. psychrophilum*.

Despite the similarity to trained immunity, the actual function of PACAP in mammalian models appears to be contrary to what has been observed in bony fish. The vast majority of mammalian studies discuss the anti-inflammatory role of PACAP during experimental bacterial infection. These experiments often involve exposure to bacterial products (such as LPS) simultaneously with PACAP, after which various immune parameters are observed (77–79). This has led to the belief that many AMPs, including PACAP, play important anti-inflammatory roles to protect the host from dangerous, over-reactive inflammatory responses (80). Though very valuable, these mammalian studies are not directly comparable to a live, growing infection within an organism or cell culture. Additionally, aside from zebrafish, teleosts appear to be lacking TLR4, which binds and responds to LPS (81, 82). As a result, observations in mammalian study systems may not be directly transferrable to those of fish. The immunostimulatory effect of PACAP on rainbow trout immune cells as observed in this study has been previously reported in head kidney leukocytes derived from another bony fish, the grass carp (71). Wang et al. (71) found that when these immune cells were exposed to bacterial products, PACAP induced inflammatory cytokine expression while having no impact on the expression of the anti-inflammatory cytokine, *IL-10*. When all of this information is taken together, it appears that PACAP may play a different, yet valuable role in the immunomodulation of teleosts when compared to what has been observed in mammals.

## Conclusions

Antimicrobial peptides (AMPs) are promising alternatives to antibiotics in the ongoing battle between aquaculture facilities and infectious agents. One AMP that has received a lot of attention due to its pleiotropic effects in aquatic species is PACAP. The results of the present study revealed that PACAP derived from the teleost *C. gariepinus* acts as a potent antimicrobial peptide against the causative agent of BCWD, *F. psychrophilum*. Furthermore, its mode of action was confirmed to be permeabilization of the bacterial membrane. When a live infection model was developed with this pathogen and the monocyte/macrophage-like cell line, RTS11, 24 h pre-exposure of PACAP appeared to protect RTS11 by significantly reducing the number of viable bacteria in the culture system. Based on transcript levels of pro-inflammatory cytokines and receptors for the AMP, PACAP was also shown to have an immunostimulatory effect on RTS11 whether exposed to the AMP alone or exposed to both PACAP and live *F. psychrophilum* challenge. Overall, this study was able to provide further validation regarding the antimicrobial effect of PACAP on aquatic pathogens as well as its immunomodulatory activity on teleost immune cells. As a promising candidate for use in aquatic models, future studies should focus on confirming these valuable functions of PACAP throughout live infection models *in vivo*.

## Author Contributions

SS performed majority of experiments, contributed to experimental design, and wrote the first draft of the manuscript. TR-R performed the remaining experiments, contributed to experimental design and aided in data/statistical analyses. YC and ME provided the synthetic PACAP and contributed to experimental design due to their previous experience studying *C. gariepinus* PACAP function on teleost growth and immunity. JL provided the strain of *F. psychrophilum* and valuable insight regarding appropriate bacterial culture/exposure conditions. BD contributed to experimental design, funding of the project, and writing of the manuscript. All authors contributed to manuscript revisions and approved the final submitted version.

### Conflict of Interest Statement

The authors declare that the research was conducted in the absence of any commercial or financial relationships that could be construed as a potential conflict of interest.

## References

[1] FAO(2018). The State of World Fisheries and Aquaculture 2018 - Meeting the Sustainable Development Goals. Rome. Licence: CC BY-NC-SA 3.0 IGO.

[2] World Bank (2014). Reducing Disease Risks in Aquaculture. World Bank Report #88257-GLB.

[3] Watts JEM Schreier HJ Lanska L Hale MS. The rising tide of antimicrobial resistance in aquaculture: sources, sinks and solutions. Marine Drug. (2017) 15:E158. 10.3390/md150601580 28587172 PMC5484108

[4] Santos L Ramos F. Antimicrobial resistance in aquaculture: current knowledge and alternatives to tackle the problem. Int J Antimicrob Agents. (2018) 52, 135–143. 10.1016/j.ijantimicag.2018.03.010 29567094

[5] Zhang L Gallo RL. Antimicrobial peptides. Curr Biol. (2016) 26, PR14–R19. 10.1016/j.cub.2015.11.017 26766224

[6] Lai Y Gallo RL. AMPed up immunity: how antimicrobial peptides have multiple roles in immune defense. Trends Immunol. (2009) 30, 131–41. 10.1016/j.it.2008.12.003 19217824 PMC2765035

[7] Lee J Luchian T Park Y. New antimicrobial peptide kills drug-resistant pathogens without detectable resistance. Oncotarget. (2018) 9:15616–34. 10.18632/oncotarget.24582 29643997 PMC5884652

[8] Mahlapuu M Hakansson J Ringstad L Bjorn C. Antimicrobial peptides: an emerging category of therapeutic agents. Front Cel Infect Microbiol. (2016) 6:194. 10.3389/fcimb.2016.00194 28083516 PMC5186781

[9] Bahar AA Ren D. Antimicrobial peptides. Pharmaceuticals. (2013) 6:1543–75. 10.3390/ph6121543 24287494 PMC3873676

[10] Kang H Kim C Seo CH Park Y. The therapeutic applications of antimicrobial peptides (AMPs): a patent review. J Microbiol. (2017) 55:1–12. 10.1007/s12275-017-6452-1 28035594

[11] Otvos L. Immunomodulatory effects of anti-microbial peptides. Acta Microbiol et Immunol Hungar. (2016) 63:257–77. 10.1556/030.63.2016.005 27539330

[12] Elssner A Duncan M Gavrilin M Wewers MD A novel P2X_7_ receptor activator, the human cathelicidin-derived peptide LL37, induces IL-1β processing and release. J Immunol. (2004) 172:4987–94. 10.4049/jimmunol.172.8.4987 15067080

[13] Yu J Mookerjee N Wee K Bowdish DME Pistolic J Li Y. Host defense peptide LL-37, in synergy with inflammatory mediator IL-1β, augments immune responses by multiple pathways. J Immunol. (2007) 179:7684–91. 10.4049/jimmunol.179.11.7684 18025214

[14] Mookherjee N Brown KL Bowdish DME Doria S Falsafi R Hokamp K. Modulation of the TLR-mediated inflammatory response by the endogenous human host defense peptide LL-37. J Immunol. (2006) 176:2455–64. 10.4049/jimmunol.176.4.2455 16456005

[15] Di Nardo A Braff MH Taylor KR Na C Granstein RD McInturff JE. Cathelicidin antimicrobial peptides block dendritic cell TLR4 activation and allergic contact sensitization. J. Immunol. (2007) 178:1829–34. 10.4049/jimmunol.178.3.1829 17237433

[16] Miyata A Arimura A Dahl RR Minamino N Uehara A Jiang L. Isolation of a novel 38 residue-hypothalamic polypeptide which stimulates adenylate cyclase in pituitary cells. Biochem Biophys Res Commun. (1989) 164:567–74. 2803320 10.1016/0006-291x(89)91757-9

[17] Kimura C Ohkubo S Ogi K Hosoya M Itoh Y Onda H A novel peptide which stimulates adenylate cyclase: molecular cloning and characterization of the ovine and human cDNAs. Biochem Biophy Res Commun. (1990) 1:81–89. 10.1016/0006-291x(90)91914-e 2302217

[18] Ogi K Kimura C Onda H Arimura A Fujino M. Molecular cloning and characterization of cDNA for the precursor of rat pituitary adenylate cyclase activating polypeptide (PACAP). Biochem Biophy Res Commun. (1990) 173:1271–9. 2268329 10.1016/s0006-291x(05)80924-6

[19] Deutsch PJ Sun Y The 38 amino acid form of pituitary adenylate cyclase activating polypeptide stimulates dual signaling cascadein PC12 cells and promotes neurite growth. J Biol Chem. (1992) 267:5108–13 1312085

[20] Matsumoto H Koyama C Sawada T Koike K Hirota K Miyake A Pituitary folliculo-stallate-like cell line (TtT/GF) responds to novel hypophysiotrophic peptide (pituitary adenylate cyclase-activating peptide), showing increased adenosine 3',5'-monophosphate and interleukin-6 secretion and cell proliferation. Endocrinology. (1993) 133:2150–5. 8404665 10.1210/endo.133.5.8404665

[21] Shivers BD Gorcs TJ Gottschall PE Arimura A. Two high affinity binding sites for pituitary adenylate cyclase-activating polypeptide have different tissue distributions. Endocrinology. (1991) 128:3055–65. 10.1210/endo-128-6-3055 2036976

[22] Pisegna JR Wank SA. Molecular cloning and functional expression of the pituitary adenylate cyclase-activating polypeptide type I receptor. Biochemistry. (1993) 90:6345–49. 8392197 10.1073/pnas.90.13.6345 PMC46925

[23] Arimura A Somogyvari-Vigh A Miyata A Mizuno K Coy DH Kitada C. Tissue distribution of PACAP as determined by RIA: highly abundant in rat brain and testes. Endocrinology. (1991) 129:2787–9. 1935809 10.1210/endo-129-5-2787

[24] Hashimoto H Ishihara T Shigemoto R Mori K Nagata S. Molecular cloning and tissue distribution of a receptor for pituitary adenylate cyclase-activating polypeptide. Neuron. (1993) 11:333–42. 10.1016/0896-6273(93)90188-W 8394723

[25] Murakami Y Koshimura K Yamauchi K Nishiki M Tanaka J Furuya H. Pituitary adenylate cyclase activating polypeptide (PACAP) stimulates growth hormone release from GH_3_ cells through type II PACAP receptor. Regul Pept. (1995) 56:35–40. 7770631 10.1016/0167-0115(95)00003-t

[26] Leyton J Gozes Y Pisegna J Coy D Purdom S Casibang M. PACAP(6-38) is a PACAP receptor antagonist for breast cancer cells. Br Cancer Res Treat. (1999) 56:177–186. 10573110 10.1023/a:1006262611290

[27] Gray SL Cummings KJ Jirik FR Sherwood NM. Targeted disruption of the pituitary adenylate cyclase-activating polypeptide gene results in early postnatal death associated with dysfunction of lipid and carbohydrate metabolism. Mol Endocrinol. (2001) 15:1739–47. 10.1210/mend.15.10.0705 11579206

[28] Adams BA Gray SL Isaac ER Biaco AC Vidal-Puig AJ Sherwood NM. Feeding and metabolism in mice lacking pituitary adenylate cyclase-activating polypeptide. Endocrinology. (2008) 149:1571–80. 10.1210/en.2007-0515 18162530 PMC2276722

[29] Starr CG Maderdrut JL He J Coy DH Wimley WC. Pituitary adenylate cyclase-activating polypeptide is a potent broad-spectrum antimicrobial peptide: structure-activity relationships. Peptides. (2018) 104:35–40. 10.1016/j.peptides.2018.04.006 29654809 PMC5982112

[30] Arimura A. Perspectives on pituitary adenylate cyclase activating polypeptide (PACAP) in the neuroendocrine, endocrine, and nervous systems. Jap J Physiol. (1998) 48:301–31. 9852340 10.2170/jjphysiol.48.301

[31] Lumsden JS Young K Welsh K MacInnes J Russell S Hesami S Management approaches for coldwater disease caused by *Flavobacterium psychrophilum*. In: Proceedings of the National Freshwater Aquaculture Symposium. (2006) p. 111–7.

[32] Starliper CE Bacterial coldwater disease of fishes caused by *Flavobacterium psychrophilum*. J Adv Res. (2011) 2:97–108. 10.1016/j.jare.2010.04.001

[33] Wiens GD Palti Y Leeds TD Three generations of selective breeding improved rainbow trout (*Oncorhynchus mykiss*) disease resistance against natural challenge with *Flavobacterium psychrophilum* during early life-stage rearing. Aquaculture. (2018) 497:414–421. 10.1016/j.aquaculture.2018.07.064

[34] Brown LL Cox WT Levine RP Evidence that the causal agent of bacterial cold-water disease *Flavobacterium psychrophilum* is transmitted within salmonid eggs. Dis Aqua Org. (1997) 29:213–8.

[35] Cipriano RC Holt RA Flavobacterium psychrophilum, Cause of Bacterial Cold-Water Disease and Rainbow Trout Fry Syndrome. Fish Disease Leaflet No. 86. Leetown, WV: United States Dept. of the Interior. U.S. Geological Service, National Fish Health Research Laboratory (2005).

[36] Nilsen H Olsen AB Vaagnes O Hellberg H Bottolfsen K Skjelstad H. Systemic *Flavobacterium psychrophilum* infection in rainbow trout, *Oncorhynchus mykiss* (Walbaum), farmed in fresh and brackish water in Norway. J Fish Dis. (2011) 34:403–8. 10.1111/j.1365-2761.2011.01249.x 21401645

[37] Silverstein JT Vallejo RL Palti Y Leeds TD Rexroad CE Welch TG Rainbow trout resistance to bacterial cold-water disease is moderately heritable and is not adversely correlated with growth. J Anim Sci. (2009) 87:860–7. 10.2527/jas.2008-1157 19028851

[38] Vallejo RL Palti Y Liu S Evenhuis JP Gao G Rexroad CE III. Detection of QTL in rainbow trout affecting survival when challenged with *Flavobacterium psychrophilum*. Mar Biotechnol. (2013) 16:349–60. 10.1007/s10126-013-9553-9 24241385

[39] Marancik D Gao G Paneru B Ma H Hernandez AG Salem M. Whole-body transcriptome of selectively bred, resistant-, control-, and susceptible-line rainbow trout following experimental challenge with *Flavobacterium psychrophilum*. Front Genet. (2015) 5:453. 10.3389/fgene.2014.00453 25620978 PMC4288049

[40] Gomez E Mendez J Cascales D Guijarro JA. *Flavobacterium psychrophilum* vaccine development: a difficult task. Microbial Biotechnol. (2013) 7:414–23. 10.1111/1751-7915.12099 25056179 PMC4229322

[41] Makesh M Sudheesh PS Cain KD. Systemic and mucosal immune response of rainbow trout to immunization with an attenuated *Flavobacterium psychrophilum* vaccine strain by different routes. Fish Shellfish Immunol. (2015) 44:156–63. 10.1016/j.fsi.2015.02.003 25687393

[42] Hoare R Ngo TPH Adams A Efficacy of a polyvalent immersion vaccine against *Flavobacterium psychrophilum* and evaluation of immune response to vaccination in rainbow trout fry (*Oncorhynchus mykiss* L.). Vet Res. (2017) 48:43 10.1186/s13567-017-0448-z 28821298 PMC5563058

[43] Nematollahi A Pasmans F Haesebrouck F Decostere A. Early interactions of *Flavobacterium psychrophilum* with macrophages of rainbow trout *Oncorhynchus mykiss*. Dis Aqua Org. (2005) 64:23–8. 10.3354/dao064023. 15900684

[44] Semple SL Mulder IM Rodriguez-Ramos T Power M Dixon B. Long-term implantation of acoustic transmitters induces chronic inflammatory cytokine expression in adult rainbow trout (*Oncorhynchus mykiss*). Vet Immunol Immunopathol. (2018) 205:1–9. 10.1016/j.vetimm.2018.10.003 30458996

[45] Ganassin RC Bols NC Development of a monocyte/macrophage-like cell line, RTS11, from rainbow trout spleen. Fish Shellfish Immunol. (1998) 8:457–76.

[46] Lugo JM Tafalla C Oliva A Pons T Oliva B Aquilino C. Evidence for antimicrobial and anticancer activity of pituitary adenylate cyclase-activating polypeptide (PACAP) from North African catfish (*Clarias gariepinus*): Its potential use as a novel therapeutic agent in fish and humans. Fish Shellfish Immunol. (2019) 86:559–70. 10.1016/j.fsi.2018.11.056 30481557

[47] Carpio Y Lugo JM Leon K Morales R Estrada MP. Novel function of recombinant pituitary adenylate cyclase-activating polypeptide as stimulator of innate immunity in African catfish (*Clarias gariepinus*) fry. Fish Shellfish Immunol. (2008) 25:439–45. 10.1016/j.fsi.2008.06.004 18652901

[48] Lugo JM Oliva A Morales A Reyes O Garay HE Herrera F. The biological role of pituitary adenylate cyclase-activating polypeptide (PACAP) in growth and feeding behavior in juvenile fish. J Pept Sci. (2010) 16:633–43. 10.1002/psc.1275 20853308

[49] Lugo JM Carpio Y Morales R Roriguez-Ramos T Ramos L Estrada MP. First report of the pituitary adenylate cyclase activating polypeptide (PACAP) in crustaceans: conservation of its functions as growth promoting factor and immunomodulator in the white shrimp *Litopenaeus vannamei*. Fish Shellfish Immunol. (2013) 35:1788–96. 10.1016/j.fsi.2013.08.028 24036332

[50] Gorgoglione B Carpio Y Secombes CJ Taylor NGH Lugo JM Estrada MP. Viral and bacterial septicaemic infections modulate the expression of PACAP splicing variants and VIP/PACAP receptors in brown trout immune organs. Fish Shellfish Immunol. (2015) 47:923–32. 10.1016/j.fsi.2015.10.014 26481517

[51] Sever L Vo NTK Bols NC Dixon B. Expression of tapasin in rainbow trout tissues and cell lines and up regulation in a monocyte/macrophage cell line (RTS11) by a viral mimic and viral infection. Devel Comp Immunol. (2014) 44:86–93. 10.1016/j.dci.2013.11.019 24321527

[52] Jarau M Di Natale A Huber P MacInnes J Lumsden JS Virulence potential of *Flavobacterium psychrophilum* isolates from Ontario, Canada in rainbow trout *Oncorhynchus mykiss* (Walbaum). J Fish Dis. (2018). 41:1505–14. 10.1111/jfd.12861 30074253

[53] Otvos L Cudic M. Broth microdilution antibacterial assay of peptides. Methods Mol Biol. (2007) 386:309–20. 10.1007/978-1-59745-430-8_12. 18604952

[54] Semple SL Kellendonk CJ Al-Hussinee L MacInnes JI Lumsden JS Dixon B Serum IgM, MH class IIβ genotype and respiratory burst activity do not differ between rainbow trout families displaying resistance or susceptibility to the coldwater pathogen, *Flavobacterium psychrophilum*. Aquaculture. (2018) 483:131–40. 10.1016/j.aquaculture.2017.10.020

[55] Lugo JM Tafalla C Leceta J Gomariz RP Estrada MP. Differential expression pattern of pituitary adenylate cyclase-activating polypeptide (PACAP) alternative splicing variants and its receptors in the immune system of rainbow trout (*Oncorhynchus mykiss*). Fish Shellfish Immunol. (2011) 30:734–8. 10.1016/j.fsi.2010.12.008 21168508

[56] Yao T Asayama Y. Animal-cell culture media: history, characteristics and current issues. Reprod Med Biol. (2017) 16:99–117. 10.1002/rmb2.12024 29259457 PMC5661806

[57] Deslouches B Phadke SM Lazarevic V Cascio M Islam K Montelaro RC. *De novo* generation of cationic antimicrobial peptides: influence in length and tryptophan substitution on antimicrobial activity. Antimicrob Agents Chemother. (2005) 49:316–22. 10.1128/AAC.49.1.316-322.2005 15616311 PMC538858

[58] Zhu X Shan A Ma Z Xu W Wang J Chou S. Bactericidal efficiency and modes of action of the novel antimicrobial peptide T9W against *Pseudomonas aeruginosa*. Antimicrobial Agents Chemother. (2015) 59:3008–17 10.1128/AAC.04830-14 25753629 PMC4432164

[59] Matsuzaki K. Control of cell selectivity of antimicrobial peptides. Biochim et Biophy Acta. (2009) 1788:1687–92. 10.1016/j.bbamem.2008.09.013 18952049

[60] Imura Y Choda N Matsuzaki K. Magainin 2 in action: distinct modes of membrane permeabilization in living bacterial and mammalian cells. Biophys J. (2008) 95:5757–65. 10.1529/biophysj.108.133488 18835901 PMC2599864

[61] Matsuzaki K. Why and how are peptide-lipid interactions utilized for self-defense? Magainins and tachyplesins as archetypes. Biochim et Biophys Acta Biomembran. (1999) 1462:1–10. 10.1016/S0005-2736(99)00197-2 10590299

[62] Huang Y Huang J Chen Y. Alpha-helical cationic antimicrobial peptides: relationships of structure and function. Protein Cell. (2010) 1:143–52. 10.1007/s13238-010-0004-3 21203984 PMC4875170

[63] Matsuzaki K Sugishita KI Fujini N Miyajima K. Molecular basis for membrane selectivity of an antimicrobial peptide, Magainin 2. Biochemicstry. (1995) 34, 3423–29. 10.1021/bi00010a034 7533538

[64] Gillooly JF Charnov EL West GB Savage VM Brown JH. Effects of size and temperature on developmental time. Nature. (2002) 417:70–3. 10.1038/417070a 11986667

[65] Patterson JT Mims SD Wright RA. Effects of body mass and water temperature on routine metabolism of American paddlefish *Polyoden spathula*. J Fish Biol. (2013) 82:1269–80. 10.1111/jfb.12066 23557305

[66] Aquilino C Granja AG Castro R Wang T Abos B Parra D. Rainbow trout CK9, a CCL25-like ancient chemokine that attracts and regulates B cells and macrophages, the main antigen presenting cells in fish. Oncotarget. (2016) 7:17547–64. 10.18632/oncotarget.8163 27003360 PMC4951232

[67] Novoa B Figueras A Ashton I Secombes CJ. *In vitro* studies on the regulation of rainbow trout (*Oncorhynchus mykiss*) macrophage respiratory burst activity. Dev Comp Immunol. (1996) 20:207–16. 8955595 10.1016/0145-305x(96)00011-0

[68] Holt RA Cytophaga Psychrophila the Causative Agent of Bacterial Cold-Water Disease in Salmonid Fish. PhD Thesis, Corvalis, OR: Oregon State University (1987).

[69] Gotlieb N Rosenne E Matzner P Shaashua L Sorski L Ben-Eliyahu S. The misleading nature of *in vitro* and *ex-vivo* findings in studying the impact of stress hormones on NK cell cytotoxicity. Brain Behav Immun. (2015) 45, 277–86. 10.1016/j.bbi.2014.12.020 25546569 PMC4342306

[70] Lugo JM Carpio Y Oliva A Morales A Estrada MP. Pituitary adenylate cyclase-activating polypeptide (PACAP): a regulator of the innate and acquired immune functions in juvenile fish. Fish Shellfish Immunol. (2010) 29:513–20. 10.1016/j.fsi.2010.05.004 20510368

[71] Wang X Wei H Zhao T Zhu X Yang X Chen D. Evidence for pituitary adenylate cyclase-activating peptide as a direct immunoregulator in teleost head kidney. Fish Shellfish Immunol. (2013) 34:265–72. 10.1016/j.fsi.2012.11.002 23153905

[72] Cardoso JCR Felix RC Martins RST Trindade M Fonseca VG Fuentes J. PACAP system evolution and its role in melanophore function in teleost fish skin. Mol Cell Enodcrinol. (2015) 411:130–45. 10.1016/j.mce.2015.04.020 25933704

[73] Sato H Feix JB. Peptide-membrane interactions and mechanisms of membrane destruction by amphipathic α-helical antimicrobial peptides. Biochim et Biophys Acta Biomembran. (2006) 1758:1245–56. 10.1016/j.bbamem.2006.02.021 16697975

[74] Kasica-Jarosz N Podlasz P Kaleczyc J. Pituitary adenylate cyclase-activating polypeptide (PACAP-38) plays an inhibitory role against inflammation induced by chemical damage to zebrafish hair cells. PLoS ONE. (2018) 13:e0198180. 10.1371/journal.pone.0198180 29856797 PMC5983416

[75] Chiou PP Khoo J Bols NC Douglas S Chen TT Effects of linear cationic α-helical antimicrobial peptides on immune-relevant genes in trout macrophages. Dev Comp Immunol. (2006) 30:797–806. 10.1016/j.dci.2005.10.011 16352337

[76] Quintin J Saeed S Martens JHA Giamarellos-Bourboulis EJ Ifrim DC Logie C. *Candida albicans* infection affords protection against reinfection via functional reprogramming of monocytes. Cell Host Microbe. (2012) 12:223–32. 10.1016/j.chom.2012.06.006 22901542 PMC3864037

[77] Delgado M Munoz-Elias EJ Gomariz RP Ganea D. VIP and PACAP inhibit IL-12 production in LPS-stimulated macrophages. Subsequent effect on IFNγ synthesis by T cells. J Neuroimmunol. (1999) 96, 167–81. 10.1016/S0165-5728(99)00023-5 10337915

[78] Delgado M Ganea D. Inhibition of endotoxin-induced macrophage chemokine production by vasoactive intestinal peptide and pituitary adenylate cyclase-activating polypeptide *in vitro* and *in vivo*. J Immunol. (2001) 167:966–75. 10.4049/jimmunol.167.2.966 11441105

[79] Martinez C Abad C Delgado M Arranz A Juarranz MG Rodriquez-Hench N. Anti-inflammatory role of septic shock of pituitary adenylate cyclase-activating polypeptide receptor. Pro Nat Acad Sci USA. (2002) 99:1053–8. 10.1073/pnas.012367999 11792830 PMC117428

[80] Delgado M Munoz-Elias EJ Gomariz RP Ganea D. Vasoactive intestinal peptide and pituitary adenylate cyclase-activating polypeptide enhance IL-10 production by murine macrophages: *in vitro* and *in vivo* studies. J Immunol. (1999) 162:1707–16. 9973433

[81] Sepulcre MP Alcaraz-Perez F Lopez-Munoz A Rocz FJ Meseguer J Cayuela ML Evolution of lipopolysaccharide (LPS) recognition and signaling: fish TLR4 does not recognize LPS and negatively regulates NF-kappaB activation. J Immunol. (2009) 182:1836–45. 10.4049/jimmunol.0801755 19201835

[82] Ali A Rexroad CE Thorgaard GH Yao J Salem M. Characterization of the rainbow trout spleen transcriptome and identification of immune-related genes. Front. Genet. (2014) 5:348. 10.3389/fgene.2014.00348 25352861 PMC4196580

